# Lifespan reduction due to neoplasia is nullified by pseudoexfoliation syndrome

**DOI:** 10.1016/j.heliyon.2018.e00832

**Published:** 2018-10-04

**Authors:** Jon Klokk Slettedal, Leiv Sandvik, Amund Ringvold

**Affiliations:** a Institute of Clinical Medicine, University of Oslo, Norway; b Department of Ophthalmology, Oslo University Hospital, Norway; c Oslo Center for Biostatistics and Epidemiology, Oslo University Hospital, Norway

**Keywords:** Epidemiology, Ophthalmology

## Abstract

**Background:**

Pseudoexfoliation syndrome (PES) is a common eye condition, indicating a risk of various eye diseases. Whether or not PES has extra-ocular physiological or even pathophysiological implications has been a matter of controversy for years.

**Methods:**

In total 1888 persons were examined for PES in 1985–86. Of these, 1864 (98·7%) had died and were therefore available for analysis by 01.01.2016. Age and cause(s) of death were recorded. 9 diagnostic groups (cardiovascular disease, cerebrovascular disease, neoplasms, systemic hypertension, diabetes mellitus (DM), chronic obstructive pulmonary disease (COPD), Parkinson's disease, aortic aneurysm (AA), and amyloidosis) based on ICD-coding were analyzed for both a possible association between PES and lifespan, as well as PES and specific systemic diseases.

**Findings:**

In the cardiovascular group, PES was not associated with an alteration in longevity. The subgroups *acute myocardial infarction* and *other cardiovascular diseases* revealed significantly reduced and increased lifespan, respectively, compared to the rest of the population. These deviations were independent of PES. The impact of PES on the neoplasm group showed that PES-positive persons lived 1·81 years (p < 0·001) longer than PES-negative persons. No significant differences in the PES prevalence were found in any of the cause of death diagnostic groups.

**Interpretations:**

The present study suggests that lifespan reduction due to neoplasia is nullified by PES, and that this phenomenon is not restricted to one specific neoplasm type. Thus, the paradoxical conclusion emerges that PES provides a lifespan benefit to the patient with a neoplasm. For the remaining diagnostic groups, PES was neither associated with an altered lifespan, nor with any cause of death diagnoses.

## 1 Introduction

Ocular PES was first described in 1917 [1]. The most striking eye changes are small grey deposits on the anterior lens surface and along the pupillary border. Over the years, a large number of studies have shown that the presence of this material should be regarded as a warning, as it may indicate serious ocular disease such as glaucoma, and can also confer an increased risk for various intra- and postoperative complications [2].

During the next five decades, PES only caught the attention of ophthalmologists. However, it transpired that similar aggregates are found both in extrabulbar connective tissue [3], and in remote tissues and organ systems [4, 5]. The aberrant material showed similarities with elastic fibrils both biochemically [6], and in electron microscopic sections [7]. These observations have been confirmed [8], and extended through the demonstrated association between the lysyl oxidase-like 1 (LOXL1) gene and PES [9], and that LOXL1 catalyzes the deamination of lysyl, which causes polymerization of tropoelastin to elastin [10]. In addition, molecular genetic studies have revealed an association between a new locus (CACNA1A rs4926244), outside of LOXL1, and PES [11].

Despite marked changes in various tissues and organs [8], population-based observations indicate that PES has no effect on all-cause mortality [12, 13]. In line with these observations, we previously found normal lifespan in persons with ocular PES [14]. Still, impact of PES on survival in certain subgroups could not be excluded. Increased risk of vascular disease has been associated with PES in some studies [15], whereas others found no association between ocular PES and cardiovascular or cerebrovascular mortality [16]. The latter authors added that all-cause mortality was significantly less in patients with ocular PES. A recent review and meta-analysis concluded that, overall, current literature suggests that PES is associated with increased risk of vascular disease [17]. There is also a disagreement as to the occurrence of PES both in diabetics and among patients with AA. Some authors claim that PES is less frequent in diabetics [18], whilst no clear association has been observed by others [19]. One report showed similar frequency of PES in patients with and without AA [20]. However, Schumacher et al. (2001) showed an increased number of PES cases in patients with AA [21]. It should be noted that the latter observation was based on an unconventional definition of PES [22].

As can be seen, to what extent ocular PES is associated with functional aberrations or systemic disease is still unclear. Therefore, the present study focuses on whether PES leads to an alteration in longevity in various systemic diseases, and on the relationship between the prevalence of ocular PES and cause of death. These issues will be addressed by reviewing the causes of death information from a population-based study with a 30-year follow-up and comparing it with PES prevalence data recorded at baseline.

## 2 Materials and methods

Data from a prospective epidemiological study conducted in three municipalities in the county of Sør-Trøndelag, Norway in 1985–86 was reviewed [23]. All inhabitants above 64 years of age (2109 persons) were invited to participate, of whom 1888 (1018 women, 870 men) were examined. Attendance rates of 84·8%, 97·7%, and 94·5% in the municipalities Hitra, Holtålen, and Rennebu, respectively were achieved. Informed consent was obtained according to the Helsinki declaration. Conventional slit-lamp examination was performed on a dilated pupil to examine for PES. Due to various ocular diseases (corneal opacities, enucleation etc.) 43 persons had only one eye examined. “PES-positive” indicated PES in at least one eye.

After approval from the Regional Ethical Committee, mortality information, including date and cause(s) of death, was obtained from The Norwegian Cause of Death Registry. Every death in Norway has to be reviewed by a medical doctor who examines the deceased person and fills in a death certificate with compulsory diagnostic coding. An autopsy is performed in a minority of cases, when death is unexpected or the cause is uncertain. As of 01.01.16, of the 1888 examined individuals, 13 were still alive, and 11 persons were recorded as deceased without any diagnostic coding. Thus, 1864 participants (98·7%) were left for analysis.

The cause of death was coded according to WHOs International Statistical Classification of Diseases and Related Health Problems (ICD) versions 8 (used 1969–85), 9 (used 1986–95) and 10 (used from 1996). 574 persons (30·1%) had a single diagnosis indicating cause of death, and the remaining 1290 persons had one diagnosis indicating the primary cause of death, and in addition one or more (up to a maximum of 9) diagnoses that were considered contributing to death. Based on the ICD-coding the deceased were classified in the following groups according to cause of death: Cardiovascular disease (with subgroups), cerebrovascular disease, neoplasms (with subgroups), systemic hypertension, DM, COPD, Parkinson's disease, AA, and amyloidosis (Table 1).

**Table 1:** Overview of ICD-coding of cause of death for systemic diseases. 23, 950, and 891 persons were coded according to ICD-8, ICD-9, and ICD-10, respectively.

Diagnostic group	Diagnostic coding system		
	ICD-8	ICD-9	ICD-10
Cardiovascular, total	393-429	391, 394-429	I01, I05-52
- Acute myocardial infarction	410, 411	410, 411	I21-24
- Other cardiovascular disease	393-404, 412-429	391, 394-405, 412-429	I01, I05-20, I25-52
Cerebrovascular	430-438	430-438	I60-69
Neoplasms	140-228	140-239	C00-D48
Systemic hypertension	400-404	401-405	I10-15
Diabetes mellitus	250	249, 250	E10-14
COPD	490-492	490-492, 494-496	J40-44, J47
Parkinson's disease	342	332	G20-21
Aortic aneurysm	441	441·1-9	I71·1-9
Amyloidosis	276	277·3	E85

### 2.1 Statistical analysis

Results on continuous variables are presented as means and standard deviations. Results on categorical variables are presented as number of subjects and percentages.

When comparing mean age at inclusion in two death diagnostic groups, e.g. persons with and without neoplasms as death diagnosis, an independent samples t-test was employed, while a chi-square test was used when comparing percentages.

When comparing mean lifespan in two death diagnostic groups, linear regression analysis was used, with ‘lifespan’ as dependent variables, and ‘death diagnostic group’ as independent variable. In order to adjust for possible differences in ‘age at baseline’ and ‘gender’ between the death diagnostic groups, these variables were also included as independent variables.

For each linear regression analysis performed, the underlying statistical assumptions were checked, and found to be adequately met.

A significance level of 5% was used. The statistical analysis was performed using IBM-SPSS version 20 software.

## 3 Results

The data was analyzed for differences in lifespan and cause of death in different categories in PES-negative (PES-) versus PES-positive (PES+) persons. Of the 1864 (1003 female, 861 male) deceased persons with registered cause of death, 317 (17·0%) showed ocular PES at examination in 1985–86. Mean age at examination was 74·1 years and 77·2 years for PES- and PES+ groups, respectively (p < 0·001), whereas mean age at death was 85·3 years and 87·1 years, respectively (p < 0·001).

### 3.1 Comparison of lifespan

Each of the ten largest death diagnostic groups (Table 2; cardiovascular disease with two subgroups, cerebrovascular disease, neoplasms, DM, systemic hypertension, COPD, Parkinson's disease, and AA) were tested for lifespan by comparing each single death diagnostic group against the remaining population, using linear regression analysis to adjust for age and gender. Subsequently, each of the eight largest groups were split into PES- and PES+, and tested accordingly.

**Table 2:** Lifespan in each diagnostic group compared to the rest of the study population, adjusted for gender and age at inclusion. The sum of persons in the second column exceeds the total population (n = 1864) because some of them had been given more than one death diagnosis. COPD = chronic obstructive pulmonary disease. * Too small groups for statistical evaluation.

Diagnostic group	Number of persons	Difference, living years (p-value)		
		Total	PES-	PES+
Cardiovascular, total	685	−0·21 (0·468)	−0·07 (0·833)	−0·94 (0·155)
- Acute myocardial infarction	484	−1·37 (<0·001)	−1·21 (<0·001)	−2·21 (0·004)
- Other cardiovascular disease	201	+2·65 (<0·001)	+2·83 (<0·001)	+1·98 (0·039)
Cerebrovascular	240	−0·81 (0·050)	−0·66 (0·151)	−1·51 (0·101)
Neoplasms	397	−1·43 (<0·001)	−1·74 (<0·001)	+0·07 (0·923)
Diabetes mellitus	164	−0·62 (0·203)	−0·42 (0·428)	−1·63 (0·172)
Syst. hypertension	138	+0·53 (0·312)	+0·63 (0·274)	−0·194 (0·881)
COPD	74	−0·01 (0·990)	−0·10 (0·893)	+0·81 (0·671)
Parkinson's disease	29	−1·80 (0·106)	*	*
Aortic aneurysm	13	+0·91 (0·583)	*	*
Amyloidosis	2	*	*	*

There was no statistical difference in lifespan of the total cardiovascular death diagnostic group versus the remaining population, nor when evaluated at the PES-/PES+ level. However, people in the subgroup “acute myocardial infarction” lived 1·37 years less than the rest of the population while people in the subgroup “other cardiovascular disease” as death diagnosis lived 2·65 years longer, with both differences being statistically significant. With respect to the PES-/PES+ level, see Table 2.

Patients in the neoplasm group (Table 2) lived significantly shorter (1·43 years) than the rest of the population. However, and interestingly, in this group (neoplasm group), PES+ persons showed a mean lifespan similar to the non-neoplasm population. This implies that people with PES and neoplasia lived 1·81 years longer than people with just neoplasia (p < 0·001).

The mean age at inclusion was 73·53 years in the neoplasm group (n = 397) versus 74·94 years in the rest of the population (n = 1467) (p < 0·001).

The following figures were found within the tumor group itself, i.e. 397 cases with neoplasm as death diagnosis: Mean age at inclusion was 73·00 years in the PES- group (n = 326) and 75·99 years in the PES+ group (n = 71) (p < 0·001). As to life span in this neoplasm group, the mean values were 83·30 years for PES- persons versus 86·80 years for PES+ persons (p < 0·001). These differences were all accounted for in the statistical evaluations.

**Fig. 1:**
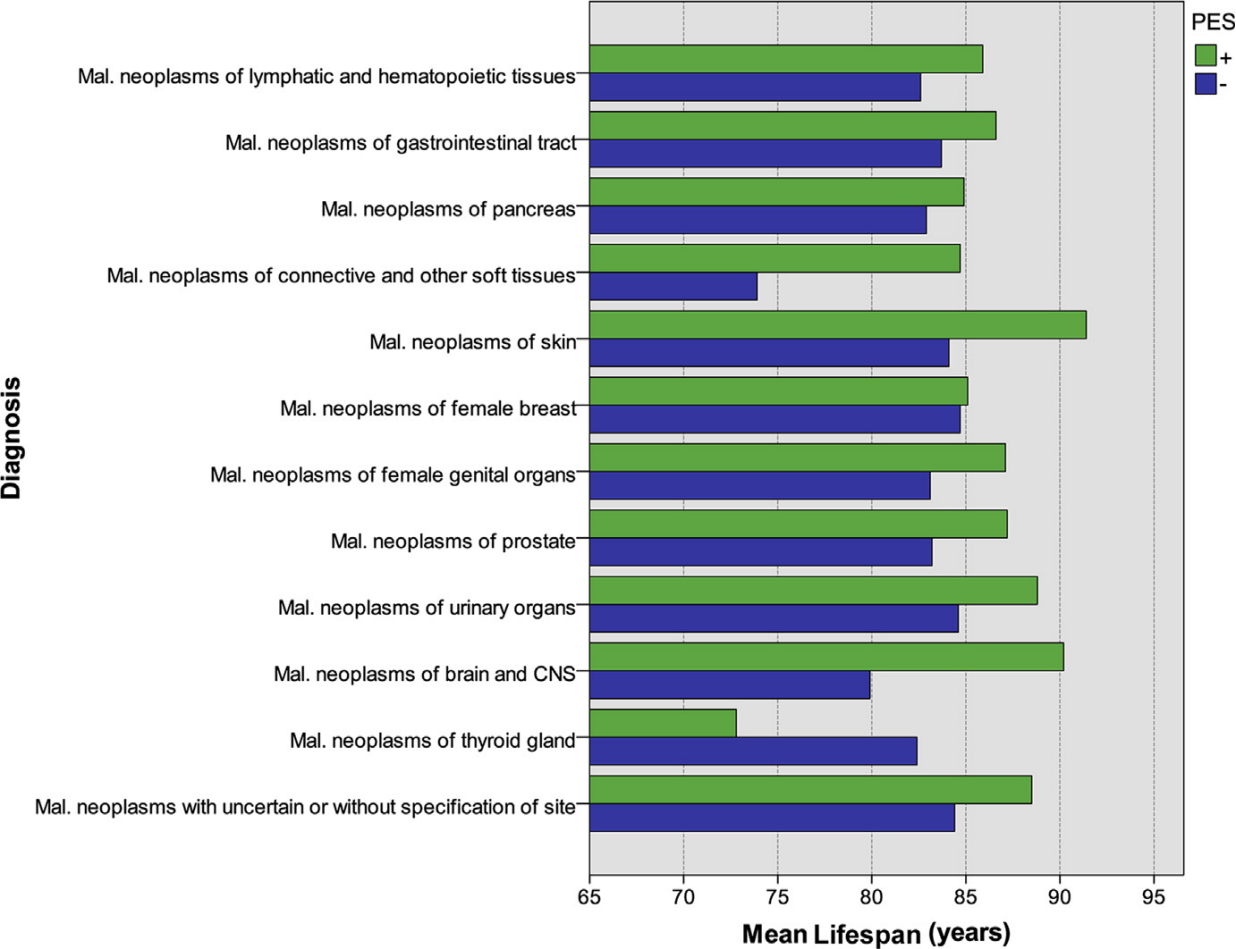
Mean lifespan in PES- and PES+ persons in various neoplasm subgroups. Only subgroups containing both PES- and PES+ persons included (i.e. 12 of 17 subgroups). For numbers of persons in each specific group, see Table 3.

Due to low numbers, the tumor subgroups were not evaluated for possible lifespan differences between PES- and PES+ cases. However, it is noteworthy (Fig. 1) that PES+ persons showed the highest mean longevity in all but one tumor subgroup (malignant neoplasms of the thyroid gland, two cases in the subgroup). Accordingly, comparing PES- numbers to the control group may indicate whether the longevity gaps between PES- and PES+ persons are statistically significant (Table 3).

**Table 3:** Lifespan of PES- persons in tumor subgroups compared to the remaining non-tumor population (n = 1467). Mal = malignant. * Too small groups for statistical evaluation.

Diagnostic subgroups of neoplasms	Total	PES- (♀/♂)	p-value
Mal. neoplasms of lymphatic and hematopoietic tissues	41	34 (13/21)	0·003
Mal. neoplasms of gastrointestinal tract	86	68 (31/37)	0·004
Mal. neoplasms of liver, gallbladder and bile ducts	7	7 (7/0)	0·09
Mal. neoplasms of pancreas	28	21 (14/7)	0·031
Mal. neoplasms of nasal cavities, middle ear and accessory sinuses	1	0 (0/0)	*
Mal. neoplasms of trachea, bronchus and lung	24	24 (2/22)	<0·001
Mal. neoplasms of connective and other soft tissues	2	1 (1/0)	*
Mal. neoplasms of skin	15	10 (5/5)	0·36
Mal. neoplasms of breast	31	26 (25/1)	0·29
Mal. neoplasms of female genital organs	23	18 (18/0)	0·062
Mal. neoplasms of prostate	76	67 (0/67)	<0·001
Mal. neoplasms of urinary organs	22	19 (6/13)	0·34
Mal. neoplasms of eye	2	2 (1/1)	*
Neoplasms of brain and CNS	7	6 (3/3)	0·024
Mal. neoplasms of thyroid gland	2	1 (1/0)	*
Mal. neoplasms with uncertain or without specification of site	26	18 (13/5)	0·31
Neoplasms of uncertain or benign behavior of digestive and respiratory systems	4	4 (3/1)	*

SUM	397	326 (143/183)	<0·001

Regarding lifespan in the cerebrovascular, DM, systemic hypertension, and COPD groups, no significant differences were observed between each group and the remaining persons, either with or without respect to PES status (Table 2). As to the Parkinson's disease, AA, and amyloidosis, see Table 2.

### 3.2 Comparison of cause of death

The number of persons in each group based on cause of death and PES prevalence is presented in Table 4. As shown, there was no statistical difference between PES- and PES+ cases concerning cause of death in any of the eight largest diagnostic groups. As to the Parkinson's disease, AA and amyloidosis, see Table 4.

**Table 4:** Cause of death in each diagnostic group compared to the rest of the study population, adjusted for gender and age at inclusion. The number of included persons were 1864, 317 (17·0%) of them had PES at examination. The diagnosis given in the left column was registered as the primary cause of death in all cases for cardiovascular and cerebrovascular cases, and for 322 (neoplasm), 44 (diabetes mellitus), 35 (systemic hypertension), 45 (COPD), 11 (Parkinson's disease), 11 (aortic aneurysm), and 0 (amyloidosis) persons for the other diagnostic groups, respectively. * Too small groups for statistical evaluation.

Diagnostic group	Number of persons	PES- (n = 1547) (%)	PES + (n = 317) (%)	P-value
Cardiovascular, total	685	574 (37·1)	111 (35·0)	0·482
- Acute myocardial infarction	484	409 (26·4)	75 (23·7)	0·304
- Other cardiovascular disease	201	165 (10·7)	36 (10·8)	0·718
Cerebrovascular	240	197 (12·7)	43 (13·6)	0·688
Neoplasms	397	326 (21·1)	71 (22·4)	0·600
Diabetes	164	140 (9·0)	24 (7·6)	0·397
Syst. hypertension	138	118 (7·6)	20 (6·3)	0·414
COPD	74	65 (4·2)	9 (2·8)	0·258
Parkinson's disease	29	25 (1·6)	4 (1·3)	*
Aortic aneurysm	13	12 (0·8)	1 (0·3)	*
Amyloidosis	2	2	0	*

However, when regarding the subgroups of neoplasms (Table 5) the results were more interesting: In total, 397 deaths (356 solid tumors and 41 hematological cancer) were caused primarily (322 persons) or partly (75 persons) by neoplasm, and the different subtypes are listed. The total neoplasm group showed PES prevalence (17·9%) similar to the overall level (17·0%). Splitting the neoplasm group into subgroups, revealed large, but not statistically significant variation of the PES prevalence. Due to low numbers, only some of the subgroups have been evaluated further. The lack of PES in the pulmonary cancer group should be noted (p = 0·018).

**Table 5:** Cause of death of neoplasms, organized in subgroups. The five largest subgroups were evaluated for possible differences between PES- and PES+, compared to the rest of the neoplasm population. Note no PES in pulmonary cancer. Mal. = malignant. * Too small groups for statistical evaluation.

Diagnostic subgroup of neoplasms	PES- and PES+	PES+ only (%)	p-values
Mal. neoplasms of lymphatic and hematopoietic tissues	41	7 (17·1)	*
Mal. neoplasms of gastrointestinal tract	86	18 (20·9)	0·610
Mal. neoplasms of liver, gallbladder and bile ducts	7	0 (0·0)	*
Mal. neoplasms of pancreas	28	7 (25·0)	0·310
Mal. neoplasms of nasal cavities, middle ear and accessory sinuses	1	1 (100·0)	*
Mal. neoplasms of trachea, bronchus and lung	24	0 (0·0)	0·018
Mal. neoplasms of connective and other soft tissues	2	1 (50·0)	*
Mal. neoplasms of skin	15	5 (33·3)	*
Mal. neoplasms of female breast	31	5 (16·1)	*
Mal. neoplasms of female genital organs	23	5 (21·7)	*
Mal. neoplasms of prostate	76	9 (11·8)	0·126
Mal. neoplasms of urinary organs	22	3 (13·6)	*
Mal. neoplasms of eye	2	0 (0·0)	*
Neoplasms of brain and CNS	7	1 (14·3)	*
Mal. neoplasms of thyroid gland	2	1 (50·0)	*
Mal. neoplasms with uncertain or without specification of site	26	8 (30·1)	0·076
Neoplasms of uncertain or benign behavior of digestive and respiratory systems	4	0 (0·0)	*

SUM	397	71 (17·9)	

## 4 Discussion

In this follow-up study, 1888 persons above 64 years underwent examination for ocular pseudoexfoliation in 1985–86. A strength of our report is that 1864 (98·7%) have been followed to death, i.e. the results are not restricted to a limited group with early death, but rather represent the actual lifespan in all cases. Furthermore, diagnostic precision in the neoplasm groups is high, due to hospitalization of these patients with biopsy verification of the diagnosis.

A shortcoming of our study is the fact that the ocular status (i.e. whether PES- or PES+) was established in 1985–86, whereas the death diagnoses were recorded during the following 30 years. As the prevalence of PES increases with age, and the development of PES is an irreversible process, there is a bias towards undetected PES+ cases being classified among the PES- group. This means that our recorded PES+ rates are underestimated when compared to rates at death. The impact of this phenomenon on lifespan calculations in our previous report [14], has been estimated to be small, with the bias unlikely to change the main results [24].

However, the final answer to this question cannot be given at present. It should also be added that the main variable affecting survival in neoplasm patients is the stage of the tumor at the time of diagnosis, and unfortunately, this information has not been available. Likewise, the low number of PES+ patients (71 cases) in the total neoplasia group (397 cases) is an important limitation.

Regarding total cardiovascular death, lifespan in this group is not significantly different compared to the rest of the population (Table 2). This conclusion remains true when PES status is taken into consideration. These results are in keeping with our previous report showing that, in the general population, lifespan is not statistically different in persons with or without PES [14].

However, splitting the total cardiovascular group (containing both PES- and PES+) into acute myocardial infarction and other cardiovascular diseases revealed a reduced and an increased lifespan in the two subgroups, respectively. The same pattern is also seen when testing for the impact of PES status on lifespan in the two subgroups (Table 2). Accordingly, these deviations in lifespan are independent of PES. This is in line with Shrum et al. [16] who found no association between ocular PES and cardiovascular or cerebrovascular mortality, and contradicts a previous report claiming ocular PES is linked to cardiovascular disease [25].

The situation is different concerning the group of patients who died due to neoplasm. As seen in Table 2, lifespan reduction due to neoplasm is nullified by PES, implying that either PES has an impact on the tumor process specifically, or that PES might have a positive impact on another system that improves lifespan. An interesting question is whether this effect refers only to specific types of neoplasm, or if it applies to malignant tumor growth in general.

An answer to these questions may to some extent be given by the two following observations: Firstly, in the neoplasm group, PES+ patients lived longer than PES- patients (Fig. 1). Secondly, lifespan in PES+ cases with neoplasm was the same as the control population (Table 2). Thus, it could be concluded that the PES compromises tumor growth generally, and that PES presence in the neoplasm group accounts for the lifespan increase.

Thus, the paradoxical conclusion emerges that PES provides a lifespan benefit to the patient with a neoplasm. It can only be speculated as to why this occurs. It is known that pseudoexfoliation material accumulates in the extracellular space, and that the extracellular matrix is essential in controlling cell invasion and determining metastatic processes [26, 27, 28]. It can be worthwhile to further examine this effect to elicit whether the PES-tumor interaction may be exploited therapeutically.

The cause of death diagnoses are equally distributed in the PES- and PES+ subgroups (Table 4), whereas lifespan is significantly reduced in persons with neoplasms (Table 2). This is taken to indicate that the cause of death diagnoses in general is not influenced by PES presence. However, once PES is present, it seems to counteract lifespan reduction in the neoplasm group. Regarding the tumor subgroups (Table 5), it is noteworthy that PES was not found in any of the 24 people who died due to pulmonary cancer. On the other hand, one study reported that COPD is associated with increased all-cause mortality in subjects with PES [13].

We found no difference in mortality rates of diabetics with and without PES (Table 4). Lifespan of the total DM group, i.e. regardless of PES-status, was not significantly reduced when compared with control. This corresponds to recent trends showing comparable life expectancy of patients with DM and the general population. There is some disagreement concerning the relationship between DM and PES. In some reports the frequency of PES, or pseudoexfoliation glaucoma, is low in diabetic patients [18], whereas others found that the occurrence of PES in diabetics is comparable to the general population [19].

Pseudoexfoliation aggregates contain abnormal elastic components [6, 7]. As the aorta and its branches are classified as elastic arteries due to the marked presence of elastic fibers in the vessel walls, it was suspected that PES+ persons could suffer from weakness in the wall of large arteries, especially the aorta [21]. It should be kept in mind, however, that the pathogenesis of AA is a multifactorial process involving degradation of not only elastin, but also collagen and other components of the aortic media and supporting laminae [29]. A clear-cut association between AA and PES has so far not been established [20, 21, 22]. In our study, 13 (10 men, 3 women) of 1864 persons died due to AA, i.e. 0·70%. According to ultra-sonographic registrations, abdominal AA is four to five times more frequent in men than in women, and in men older than 65, ruptured abdominal AA are responsible for 2·1% of all deaths [30]. A final conclusion concerning the relationship between AA and PES cannot be drawn from our figures, because abdominal ultra-sonography has not been performed.

In conclusion, our main finding is that PES appears to benefit the person suffering from a neoplasm. This observation needs to be further studied.

## Declarations

### Author contribution statement

Jon Klokk Slettedal, Amund Ringvold: Conceived and designed the experiments; Performed the experiments; Analyzed and interpreted the data; Contributed reagents, materials, analysis tools or data; Wrote the paper.

Leiv Sandvik: Conceived and designed the experiments; Performed the experiments; Analyzed and interpreted the data.

### Funding statement

This work was supported by The Norwegian Society of the Blind and Partially Sighted, The Raagholt Research Foundation, and Institute of Clinical Medicine, University of Oslo.

### Competing interest statement

The authors declare no conflict of interest.

### Additional information

No additional information is available for this paper.

## References

[1] Lindberg J.G. (1989). Clinical investigations on depigmentation of the pupillary border and translucency of the iris in cases of senile cataract and in normal eyes in elderly persons (1917). Acta Ophthalmol..

[2] Drolsum L., Ringvold A., Nicolaissen B. (2007). Cataract and glaucoma surgery in pseudoexfoliation syndrome: a review. Acta Ophthalmol. Scand..

[3] Ringvold A. (1973). On the occurrence of pseudo-exfoliation material in extrabulbar tissue from patients with pseudo-exfoliation syndrome of the eye. Acta Ophthalmol (Copenh).

[4] Sugino T. (1990). Exfoliation materials in the skin of patients with exfoliation syndrome. Nippon. Ganka Gakkai Zasshi.

[5] Schlötzer-Schrehardt U.M., Koca M.R., Naumann G.O.H., Volkholz H. (1992). Pseudoexfoliation syndrome. Ocular manifestation of a systemic disorder?. Arch. Ophthalmol..

[6] Ringvold A. (1973). A preliminary report on the amino acid composition of the pseudoexfoliation material (PE material). Exp. Eye Res..

[7] Streeten B.W., Gibson S.A., Dark A.J. (1986). Pseudoexfoliative material contains an elastic microfibrillar-associated glycoprotein. Trans. Am. Ophthalmol. Soc..

[8] Ritch R., Schlötzer-Schrehardt U. (2001). Exfoliation syndrome. Surv. Ophthalmol..

[9] Thorleifsson G., Magnusson K.P., Sulem P. (2007). Common sequence variants in the LOXL1 gene confer susceptibility to exfoliation glaucoma. Science.

[10] Liu X., Zhao Y., Gao J. (2004). Elastic fiber homeostasis requires lysyl oxidase-like 1 protein. Nat. Genet..

[11] Aung T., Ozaki M., Mizoguchi T. (2015). A common variant mapping to CACNA1A is associated with susceptibility to exfoliation syndrome. Nat. Genet..

[12] Ringvold A., Blika S., Sandvik L. (1997). Pseudo-exfoliation and mortality. Acta Ophthalmol. Scand..

[13] Svensson R., Ekström C. (2015). Pseudoexfoliation and mortality: a population-based 30-year follow-up study. Acta Ophthalmol..

[14] Slettedal J.K., Sandvik L., Ringvold A. (2015). Ocular pseudoexfoliation syndrome and life span. EBio Med..

[15] Mitchell P., Wang J.J., Smith W. (1997). Association of pseudoexfoliation syndrome with increased vascular risk. Am. J. Ophthalmol..

[16] Shrum K.R., Hattenhauer M.G., Hodge D. (2000). Cardiovascular and cerebrovascular mortality associated with ocular pseudoexfoliation. Am. J. Ophthalmol..

[17] Wang W., He M., Zhou M., Zhang X. (2014). Ocular pseudoexfoliation syndrome and vascular disease: a systematic review and meta-analysis. PloS One.

[18] Tarkkanen A., Reunanen A., Kivelä T. (2008). Frequency of systemic vascular diseases in patients with primary open-angle glaucoma and exfoliation glaucoma. Acta Ophthalmol..

[19] Allingham R.R., Loftsdottir M., Gottfredsdottir M.S. (2001). Pseudoexfoliation syndrome in Icelandic families. Br. J. Ophthalmol..

[20] Hietanen J., Soisalon-Soininen S., Kivelä T., Tarkkanen A. (2002). Evaluation of the clinical association between exfoliation syndrome and abdominal aortic aneurysm. Acta Ophthalmol. Scand..

[21] Schumacher S., Schlötzer-Schrehardt U., Martus P., Lang W., Naumann G.O.H. (2001). Pseudoexfoliation syndrome and aneurysms of the abdominal aorta. Lancet.

[22] Ringvold A. (2001). Pseudoexfoliation and aortic aneurysms. Lancet.

[23] Ringvold A., Blika S., Elsås T. (1988). The Middle-Norway eye-screening study. I. Epidemiology of the pseudo-exfoliation syndrome. Acta Ophthalmol (Copenh).

[24] Kivelä T. (2015). Ocular pseudoexfoliation syndrome and life span: Act 2. EBio Med..

[25] Siordia J.A., Franco J., Golden T.R., Dar B. (2016). Ocular pseudoexfoliation syndrome linkage to cardiovascular disease. Curr. Cardiol. Rep..

[26] Joyce J.A., Pollard J.W. (2009). Microenvironmental regulation of metastasis. Nat. Rev. Cancer.

[27] Zhu J., Liang L., Jiao Y., Liu L. (2015). On behalf of the U.S.-China Physical Sciences-Oncology Alliance. Enhanced invasion of metastatic cancer cells via extracellular matrix interface. PloS One.

[28] Multhaupt H.A.B., Leitinger B., Gullberg D., Couchman J.R. (2016). Extracellular matrix component signaling in cancer. Adv. Drug Deliv. Rev..

[29] Ailawadi G., Eliason J.L., Upchurch G.R. (2003). Current concepts in the pathogenesis of abdominal aortic aneurysm. J. Vasc. Surg..

[30] Ashton H.A., Buxton M.J., Day N.E. (2002). The multicentre aneurysm screening study (MASS) into the effect of abdominal aortic aneurysm screening on mortality in men: a randomized controlled trial. Lancet.

